# Thermally Stable Solution Processed Vanadium Oxide as a Hole Extraction Layer in Organic Solar Cells

**DOI:** 10.3390/ma9040235

**Published:** 2016-03-25

**Authors:** Abdullah Alsulami, Jonathan Griffin, Rania Alqurashi, Hunan Yi, Ahmed Iraqi, David Lidzey, Alastair Buckley

**Affiliations:** 1 Department of Physics & Astronomy, University of Sheffield, Hicks Building, Hounsfield Rd., Sheffield, South Yorkshire S3 7RH, UK; asalsulami1@sheffield.ac.uk (A.A.); jon.griffin@sheffield.ac.uk (J.G.); ralqurashi2@sheffield.ac.uk (R.A.); d.g.lidzey@sheffield.ac.uk (D.L.); 2 Department of Chemistry, University of Sheffield, Sheffield, South Yorkshire S3 7HF, UK; h.yi@ossila.com (H.Y.); a.iraqi@sheffield.ac.uk (A.I.)

**Keywords:** organic photovoltaic, vanadium oxide, thermal stability, solution processing, photoelectron spectroscopy

## Abstract

Low-temperature solution-processable vanadium oxide (V_2_O*_x_*) thin films have been employed as hole extraction layers (HELs) in polymer bulk heterojunction solar cells. V_2_O*_x_* films were fabricated in air by spin-coating vanadium(V) oxytriisopropoxide (s-V_2_O*_x_*) at room temperature without the need for further thermal annealing. The deposited vanadium(V) oxytriisopropoxide film undergoes hydrolysis in air, converting to V_2_O*_x_* with optical and electronic properties comparable to vacuum-deposited V_2_O_5_. When s-V_2_O*_x_* thin films were annealed in air at temperatures of 100 °C and 200 °C, OPV devices showed similar results with good thermal stability and better light transparency. Annealing at 300 °C and 400 °C resulted in a power conversion efficiency (PCE) of 5% with a decrement approximately 15% lower than that of unannealed films; this is due to the relative decrease in the shunt resistance (R_sh_) and an increase in the series resistance (R_s_) related to changes in the oxidation state of vanadium.

## 1. Introduction

Currently, the high cost of commercial inorganic photovoltaics remains an obstacle for the wide-scale installation in both residential and commercial settings [1]. In order to overcome this high cost, researchers have studied numerous materials as alternatives, with organic polymer solar cells being a promising candidate [2,3]. This is due to organic polymers exhibiting several advantageous properties, such as solution-processability, mechanical-flexibility, and thin device architectures allowing for light-weight devices [4,5,6]. This combination of factors allows organic solar to scale up manufacture via roll-to-roll- or sheet-to-sheet-based deposition techniques leading to reduced fabrication costs. Recent advances in polymer synthesis and device processing have pushed the power conversion efficiency of single junction cells as high as 10.8% [7]. These efficiencies are above the 10% mark often quoted as the point at which polymer photovoltaics will become commercially viable. However, currently, the lifetime of devices incorporating the highest performing materials are below the 10 year mark required; one method to improve the lifetime of devices is to replace the commonly-used hole extraction interfacial layer (HELs), polyethylene dioxythiophene:polystyrenesulfonate (PEDOT:PSS) [8,9,10]. This is because, despite the positive aspects of aqueous PEDOT:PSS, the residual moisture and the acidic nature of PEDOT:PSS can cause degradation of the electrode and organic films and, therefore, reduce the devices operational lifetime [11,12,13,14]. To overcome some of these issues thin metal oxides such as MoO_3_, WO_3_, NiO, and V_2_O_5_ have been used; these materials have been shown to exhibit performances that are similar to or better than OPVs with an interfacial PEDOT:PSS layer [15,16,17,18]. Not only are these materials of interest in organic photovoltaics because of their unique electronic properties and chemical stability, they are also being used in other photovoltaic technologies such as CiGS, and CdTe [19,20,21,22]. However, like PEDOT:PSS the presence of water can lead to changes at the interface between metal oxides and organic semiconductors, due to the intercalation of water within metal oxide clusters leading to changes within the energy level structure [23]. This makes the improvement of processing conditions and reduction in the cost and complexity of depositing these interfacial layers highly desirable.

Solution-processing of the metal oxide interfacial layers has shown significant promise for reducing the complexity of the deposition of these materials. Previous studies have shown that vanadium oxide can be deposited from solution at low temperature in air without the need for any high temperature post-deposition treatments [24,25,26,27,28]. On the other hand, some optoelectronic devices which are fabricated at high temperatures require developed metal oxides which must be able to keep their properties under high temperature fabrication processes. Whilst the optical and chemical properties of V_2_O*_x_* have previously been investigated after high-temperature annealing [29,30,31], its performance with OPVs has not. Therefore, our motivation for this work is to explore the impact of thermal heating on V_2_O*_x_* thin films as HELs in organic solar cells.

In this paper we incorporated V_2_O*_x_* thin films deposited from a vanadium(V) oxytriisopropoxide precursor into PFDT2BT-8:PC_70_BM OPV devices achieving a power conversion efficiency of 6%, comparable to PEDOT:PSS and vacuum deposited MoO_3_. Furthermore, we fabricated some OPV devices with annealed V_2_O*_x_* layers at high temperatures in air before spin coating the active layer to study their thermal stability. We demonstrated that OPV devices show PCE ≥ 5% with thermally-annealed V_2_O*_x_* at 400 °C. Absorption spectroscopy, X-ray photoelectron spectroscopy (XPS), and ultraviolet photoelectron spectroscopy (UPS) are combined to explain the J–V characteristics of OPVs.

## 2. Results and Discussion

### 2.1. PFDT2BT-8 Structure

**Figure 1:**
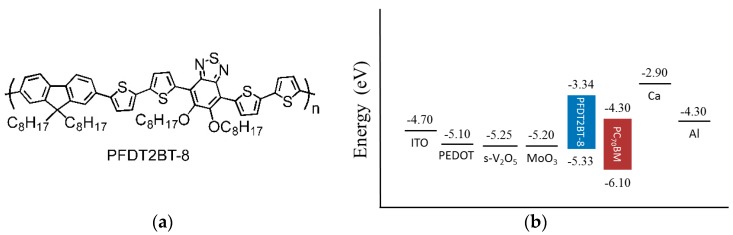
(**a**) The chemical structure of PFDT2BT-8; and (**b**) energy levels of electrodes, HELs, and active layer materials used in this work.

The chemical structure of our donor polymer used in this work is shown in Figure 1a [32]. The highest occupied molecular orbital (HOMO) energy level and lowest unoccupied molecular orbital (LUMO) of PFDT2BT-8 are −5.33 eV and −3.34 eV, respectively, as determined from a cyclic voltammetry. The energy band diagram and work function of the relative materials in our study are presented in Figure 1b [32,33].

### 2.2. s-V_2_O_x_ as a Hole Extraction Layer

To assess the performance of PFDT2BT-8 polymer with the s-V_2_O*_x_* interlayer, we fabricated sets of PFDT2BT-8:PC_70_BM devices with variable thicknesses of s-V_2_O*_x_*. The extracted data suggests an optimum s-V_2_O*_x_* layer thickness below 10 nm. Therefore, we selected a layer thickness of 5 nm for all s-V_2_O*_x_* devices.

**Figure 2:**
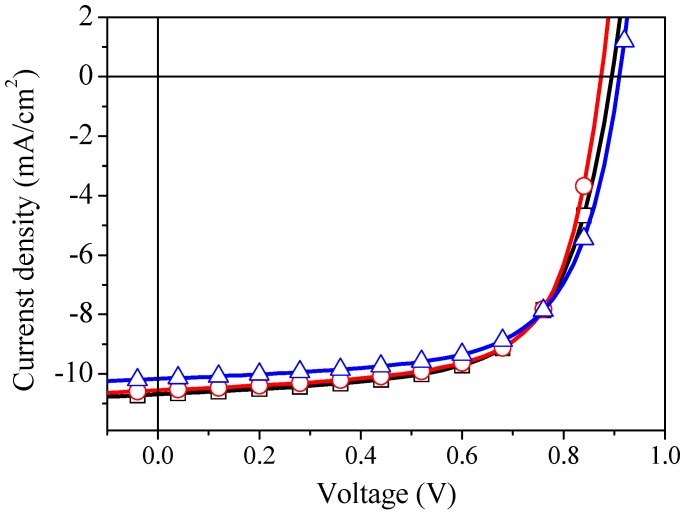
The current density-voltage characteristics of PFDT2BT-8:PC70BM based solar cell with (□) PEDOT:PSS, (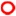) s-V2Ox, and (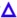) MoO_3_.

Figure 2 shows the J–V characteristics of our OPV devices and those of the most widely used HELs, PEDOT:PSS and thermally evaporated MoO_3_ under AM 1.5 G illumination. It can be seen that using PEDOT:PSS and s-V_2_O*_x_* layers as HEL showed a similar photovoltaic response (PCE = 6.5%) which is better than devices fabricated with MoO_3_ interlayer (PCE = 6.3%). These results indicate that a high performance can be achieved for PFDT2BT-8 devices by using untreated s-V_2_O*_x_* films which are in good agreement with the previous studies in literature [25,27,34].

**Figure 3:**
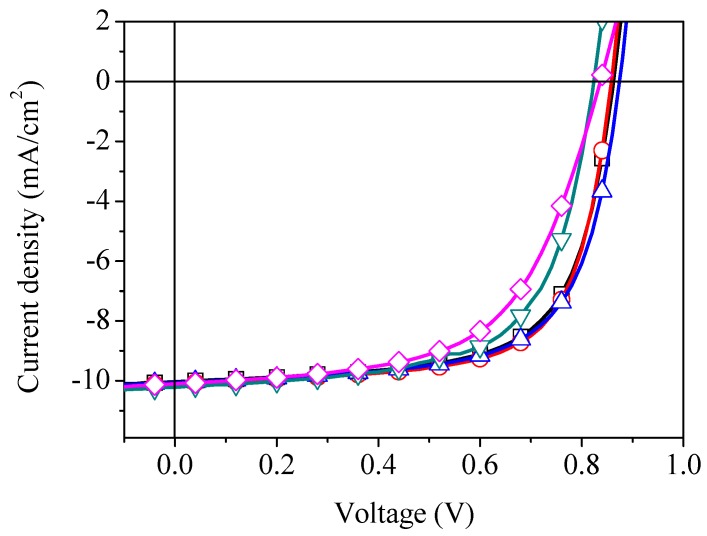
The current density-voltage characteristics of organic solar cell with (**□**) unannealed s-V_2_O*_x_* interlayer and annealed at (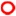) 100 °C, (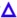) 200 °C, (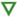) 300 °C, and (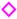) 400 °C for 30 min.

To explore the effect of thermal annealing (≥100 °C) on the photovoltaic response of our devices, s-V_2_O*_x_* films were annealed in air at temperatures of 100, 200, 300, and 400 °C for 30 min before spin coating the active layer. The corresponding current-voltage characteristic of OPVs is shown in Figure 3. The photovoltaic parameters obtained are summarised in Table 1 which represent the average of at least 12 pixels from 18 pixels defined on three separate substrates. The errors quoted are defined by the standard deviation about the mean.

It can be seen that the performance of devices annealed at temperatures 100 or 200 °C is quite similar to those prepared without heat treatment. The slight increase in PCE of devices annealed up to 200 °C can be ascribed to the relative increase in the shunt resistance R_sh_ and decrease in the series resistance R_s_. In contrast, as the film annealing temperature is raised to 400 °C, OPV devices show a PCE of 5% with a decrement approximately 15% lower than that of those with unannealed films. It is clear from Table 1 that the relatively weak photovoltaic response can be attributed to the decrease in open-circuit voltage, due to the change in chemical structure as will be discussed based on the XPS data later on. Furthermore, the shunt resistance falls considerably on annealing above 300 °C reaching a minimum R_sh_ of 933 Ω·cm^2^ on annealing at 400 °C due to the recombination process at the anode interface. This caused a decrease in hole density at the anode interface leading to a decrease in the internal electric field [35,36].

### 2.3. Optical Properties

**Figure 4:**
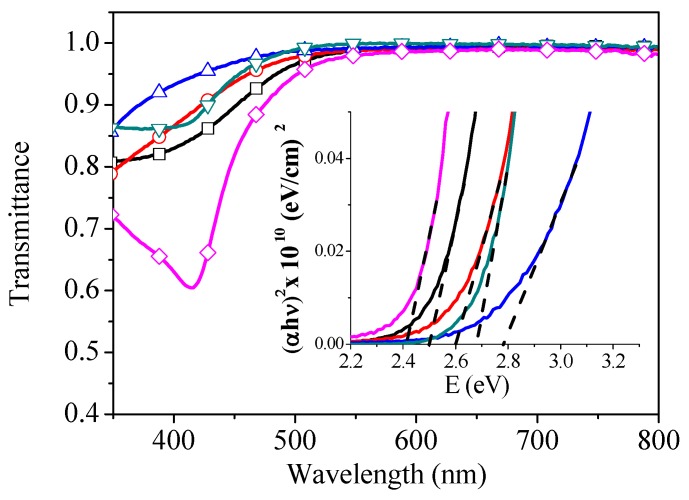
Optical transmission spectra of the s-V_2_O*_x_* films with different Annealing temperature; (**□**) unannealed, (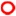) 100 °C, (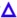) 200 °C, (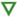) 300 °C, and (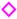) 400 °C. The insert shows the absorption coefficient (αhν)^2^ as a function of the photon energy.

To understand the changes that we observed in the photovoltaic response as a function of the annealing temperature, we investigated the optical properties of annealed s-V_2_O*_x_* films. Figure 4 presents the optical transmittance of the films as a function of the wavelength for the as-deposited and annealed films. All s-V_2_O*_x_* films show high transmission for wavelengths above 500 nm covering the majority of the solar spectrum. The transmittance in the visible wavelength range, from 400 to 500 nm, increases slightly by annealing up to 300 °C which is attributed to change of the films refractive index related to chemical structure changes. On annealing to 400 °C, the film exhibits absorption peak at 415 nm which could be ascribed to small polaron absorption [37,38]. This effect results from disordering defects in V_2_O*_x_* structure leading to transferring charges between neighbouring sites with a significant spread in energy.

Using Tauc’s Law, the optical band gap of s-V_2_O*_x_* films was determined [25,27]. The relationship between the band gap energy ($E_{g}$) and the absorption coefficient (α) can be represented as:

(1) α(hν)∝[hν− Eg]1N

where hν represents the photon energy and N is equal to 2. As can be seen in Figure 4, the optical absorption coefficient ${\left(\alpha h\nu \right)}^{2}\;$is plotted as a function of the incident photon energy. By extrapolating from the straight-line portion of the plots to the energy-axis, we determined that $E_{g}$ of the unannealed film lies at 2.5 eV which is very close to those reported in literature for solution-processed samples [24,27,39]. Several studies have reported various values of V_2_O*_x_* band gap due to the different processing conditions or the measurement method. For instance, electron spectroscopy measurements of V_2_O*_x_* layer spin coated in air from isopropanol solution of vanadium(V) oxitriisopropoxide showed a band gap of 3.6 eV [24]. In contrast, the optical absorption experiments of the same precursor revealed a lower band gap of 2.3 eV [25]. In addition, impact of the processing conditions on the V_2_O_x_ band gap has been reported by other authors [24,27,39,40,41]. For example, V_2_O*_x_* samples spin coated and annealed under N_2_ atmosphere had a band gap of 3.2 eV while fabrication of the films in air showed lower band gaps of 2.5 eV [40]. On annealing s-V_2_O*_x_* films to 200 °C, the band gap energy rises to 2.78 eV. On further annealing, $E_{g}$ reduces again to 2.42 eV at an annealing temperature of 400 °C. Looking at the individual values of J_sc_ in Table 1 it can be observed that the variation in the band gap is independent of the OPV performance.

### 2.4. Atomic Force Microscopy

**Figure 5:**
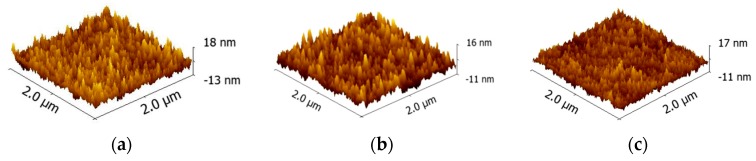
AFM topography (2 µm × 2 µm) of a 10 nm thick s-V_2_O*_x_* layer deposited on top of Si with native oxide; (**a**) as deposited; (**b**) annealed at 200 °C; (**c**) annealed at 400 °C. The average grain size was 21.3 nm, 16.8 nm, and 19.6 nm, respectively.

AFM scanning was performed to investigate the impact of thermal annealing on surface topography of s-V_2_O*_x_*. Figure 5 shows 2 µm × 2 µm AFM scans for an unannealed film (a), and films thermally-annealed at 200 °C and 400 °C for 30 min ((b) and (c), respectively). RMS roughness of films decreased slightly upon annealing, from 2.5 nm for an as-cast precursor film to 1.9 nm for films annealed at 400 °C, with no local crystallisation structure observed. The low RMS of V_2_O*_x_* annealed samples avoided short circuit faults when they were incorporated into OPV devices as shown in Table 1.

### 2.5. X-ray Photoelectron Spectroscopy

**Figure 6:**
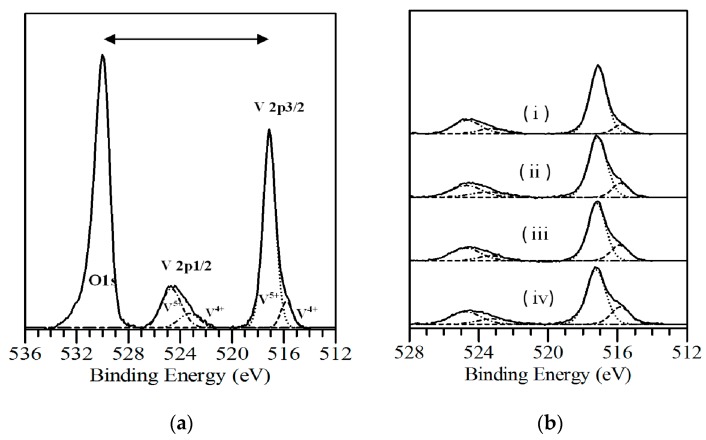
XPS spectra of s-V_2_O*_x_* thin film deposited in air. (**a**) The solid line represents the experimental XPS spectra; the dashed lines are decomposed XPS; the binding energy of V^5+^ peaks are higher than V^4+^ peaks (**b**) Photoelectron spectra of (i) unannealed film and those annealed at (ii) 200 °C; (iii) 300 °C, and (iv) 400 °C.

We investigated the chemical composition and electronic structure of s-V_2_O*_x_* thin films by photoelectron spectroscopy (UPS and XPS). Figure 6a shows the XPS spectrum of an as-cast precursor film prepared in ambient conditions. The O1s signal, determined at 530.0 eV as reported in literature [42], was used as a binding energy reference. Therefore, we find V2p_1/2_, and V2p_3/2_ lines correspond to 517.1 eV and 524.6 eV, respectively, which is in good agreement with the commonly reported values [42,43,44,45,46].

Decomposition analysis reveals that V2p_3/2_ peak consists of two different species (*i.e.*, V^5+^ and V^4+^ oxidation state) with a ratio of 5.7:1. The peak position Of V^4+^ and V^5+^ in V2p_3/2_ spectrum was identified by using L-G fitting to be 515.8 eV and 517.1 eV, respectively, in which V^4+^ oxidation state arises as a low-energy shoulder in the core level of V2p_3/2_. The calculated composition analyses indicate that a small amount of oxygen vacancy exists in unannealed s-V_2_O*_x_* films. Fabrication conditions such as atmospheric gases and thermal annealing can increase the concentration of V^4+^ species leading to the reduction of the V^5+^ to V^4+^. For instance, H_2_O molecules in air facilitate the oxygen removal such that oxygen-hydrogen interaction weakens the binding energy of the oxygen atom with the neighbouring vanadium atoms [47,48]. Moreover, a previous study demonstrated that V_2_O_5_ can be reduced during XPS measuring in which X-rays create vanadyl (V=O) oxygen atoms vacancies [49].

Figure 6b exhibits a comparison between the XPS spectra of (i) unannealed s-V_2_O*_x_* films and those annealed in air at three different temperatures (ii) 200 °C, (iii) 300 °C, and (iv) 400 °C. As observed, the binding energies of the V2p_3/2_ and O1s core levels do not change appreciably with increasing temperature. Furthermore, as the annealing temperature increases, the V 2p_3/2_ spectrum becomes broader at low binding energies due to the increase of V^4+^ species. Table 1 summarises the change of the oxidation state of vanadium with annealing temperature which is consistent with previous studies [50,51]. By deconvolution of V2p_3/2_ lines, we did not observe any shift in the binding energy of V^4+^ and V^5+^ peaks in any cases. In addition, it is evident that V^4+^ oxidation state rises steadily with increasing annealing temperatures. Therefore, V_2_O*_x_* thin films are gradually reduced owing to the generation of more oxygen vacancies. This partial reduction leads to an increase in the V3d electron density [52]. Furthermore, oxygen vacancies in V_2_O*_x_* can cause localisation excess electrons in unfilled 3d orbitals which can explain the absorption peak near to the ultraviolet band observed in Figure 4 [37]. From Table 2 we conclude that vanadium oxide can be partially reduced by a thermal annealing process in air for 30 min from V_2_O_4.93_ to V_6_O_14_. Increasing the annealing temperature can result in further reduction as reported previously [50].

In the literature, there have been detailed investigations on the physical properties and gradual reduction of vanadium oxide by heating at high temperatures [29,50].

### 2.6. Ultraviolet Photoelectron Spectroscopy

**Figure 7:**
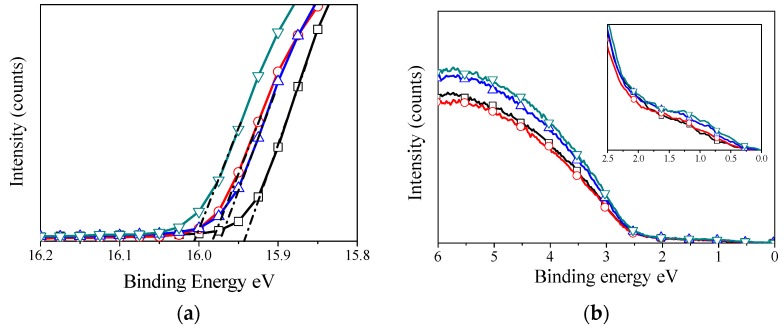
UPS measurements for V_2_O*_x_* films Span coated on ITO in ambient conditions with no annealing and annealed, (**□**) unannealed, (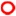) 200 °C, (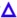) 300 °C, and (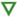) 400 °C. (**a**) shows the secondary electron cut-off region; and (**b**) shows the expanded region near the Fermi level; the insert shows the density of gap states formed about 1 eV below the Fermi level.

Figure 7 plots the UPS spectra of V_2_O*_x_* films that have been annealed at different temperatures. The work function and valence band edge determined by the UPS are also summarised in Table 1. As shown in Figure 7a, the position of the secondary electron cut-off for unannealed film was determined to be at the binding energy of 15.94 eV below the Fermi level, corresponding to a work function of 5.26 eV. Compared to previous studies, the work function of our s-V_2_O*_x_* films is similar to those prepared by different processes [34]. On annealing, the secondary electron cut-off shows a slight shift up to 0.07 eV toward the higher binding energy. While we see a 0.07 eV change in the work function we can’t assign these changes directly to the processing of the s-V_2_O*_x_* due to the possible influence of adsorbates. Figure 7b displays the onset of the s-V_2_O*_x_* valence band to be 2.5 eV below the Fermi level which is identical with all samples. We did not notice any shift for the valence band maximum with increase annealing temperature. Careful observation of the valence band region of films annealed at 300 °C and 400 °C reveals the formation of more gap states at 1 eV below the Fermi level as shown in the inset of Figure 7b. This suggests that oxygen vacancies shift the Fermi level up toward the conduction band leaving some occupied states within the band gap. Therefore, introducing oxygen vacancies can act as n-type dopants resulting in a decrease in the work function of vanadium oxide [53]. Consequently, photoelectron spectroscopy analysis confirms that the high-temperature annealing of s-V_2_O_x_ film produces a change in the chemical structure of the vanadium oxide layer and, thus, increases slightly the hole-electron recombination at the interface. Nevertheless, our experiments demonstrate that s-V_2_O*_x_* film can be used to replace evaporated metal oxides in optoelectronic devices which are fabricated at high temperatures.

## 3. Materials and Methods

### 3.1. Materials

Vanadium(V) oxytriisopropoxide was purchased from Sigma-Aldrich (Dorset, UK) and was mixed with iso-propanol (99.5%) at a 1:250 volume ratio to obtain a solution with a concentration of 4 mg·mL^−1^. PEDOT:PSS was purchased from Ossila Ltd., Sheffield, UK, MoO_3_ (99.95%) was purchased from Testbourne Ltd (Basingstoke, UK), aluminium (99.99%) and calcium (99%) were purchased from Sigma-Aldrich (Dorset, UK). The donor polymer PFDT2BT-8 was synthesised in the Department of Chemistry at the University of Sheffield via a previously reported method, and had a molecular weight of 91.6 kDa and a polydispersity index (PDI) of 1.47 [32]. PC_70_BM was purchased from Ossila Ltd with a purity of 95% (5% PC_60_BM). The active layer solution was prepared by mixing PFDT2BT-8 and PC_70_BM at a weight ratio of 1:4 in chloroform with an overall concentration of 20 mg·mL^−1^.

### 3.2. OPV Device Fabrication

All OPV devices were fabricated onto pre-patterned ITO coated glass substrates purchased from Ossila Ltd (Sheffield, UK). Prior to use, the substrates were sonicated in a warm cleaning solution of Hellmanex (2 wt %) for 10 min at 70 °C. After sonication, they were washed with the de-ionized water. They were then placed in isopropanol and sonicated for 10 min at 70 °C. Finally, they were dried with nitrogen gas. Thin films (~5 nm) of s-V_2_O*_x_* were deposited via spin coating onto cleaned ITO substrates in ambient conditions and were then transferred into a dry nitrogen glove box. A thermal evaporated MoO_3_ film (10 nm) was deposited at a rate of 3 Å·s^−1^ and at a pressure of ~10^−7^ mbar in a high-vacuum system in the glove box. PEDOT:PSS film with (30 nm ± 3 nm) thick was spin cast in ambient conditions and was then annealed at 130 °C for an hour in the glove box environment. Semiconducting thin films were prepared by spin casting the solution onto a substrate at a spin speed of 3000 rpm in order to obtain an active layer with a thickness of 70 nm ± 4 nm. The substrates were then transferred within the glove box to a high-vacuum system (10^−7^ mbar) to thermally evaporate the cathodes. The bi-layer cathodes of Ca (5 nm) and Al (100 nm) were evaporated at a rate of 3 Å·s^−1^ and 10 Å·s^−1^, respectively. The final step in device fabrication was encapsulation of the central area of each substrate by using a glass slide and light-curable epoxy, to extend the lifetime for measurement and storage.

### 3.3. Current-Density Characterisation

OPV devices were measured under ambient conditions using a Keithley 2400 source meter (Tektronix Ltd., Bracknell, UK) and a Newport 92251A-1000 AM1.5 solar simulator (Newport Co.,LTD, Didcot, UK). An NREL calibrated silicon diode was used to calibrate the power output at 1000 W·cm^−2^. Each device had an area of approximately 2.6 mm^2^ as defined by a shadow mask.

### 3.4. Atomic Force Microscopy

A Veeco Instruments Dimension 3100 was used to perform the AFM. Aluminium-coated silicon tips from Budget Sensors (Tap 300 Al-G) with a resonance frequency of 300 kHz and a spring constant of 40 N·m^−1^ were used throughout. The obtained data were processed using the Gwyddion.

### 3.5. Photoelectron Spectroscopy

The UPS and XPS measurements were carried out using KratosUltra AXIS photoelectron spectroscopy (Kratos Analytical Ltd., Manchester, UK). XPS measurements were taken using the Al Kα radiation with an energy of 1486.6 eV as the excitation source, a band pass energy of 10 eV, a step size of 0.025 eV, and a dwell time of 250 ms. UPS measurements were taken using the He (I) emission line (21.2 eV), a band pass energy of 10 eV, a step size of 0.025 eV, and a dwell time of 250 ms.

### 3.6. Absorption Spectroscopy

Absorption measurements were conducted under ambient conditions using a Horiba Fluoromax 4 spectrometer (HORIBA Ltd., Stanmore Middlesex, UK) with a xenon arc lamp (200 to 950 nm). Transmittance and absorbance of all the samples in this work were measured with the range 300–900 nm in increments of 2 nm and a slit size of 2 nm. Transmittance spectra were normalised against the power output recorded by the reference detector to account for any potential variations in light output between the separate measurements.

## 4. Conclusions

In contrast to previous studies, we have demonstrated that thermal annealing of V_2_O_x_ thin films deposited from a vanadium(V) oxytriisopropoxide does not significantly enhance the performance of OPV devices. However, the efficiency of OPV devices is reduced by 15% after annealing s-V_2_O*_x_* layers at high temperatures (*i.e.*, 400 °C) due to the decrease of V_oc_ and shunt resistance. Absorption spectroscopy studies reveal that OPV performance is independent of the variable optical band gap of s-V_2_O*_x_*. XPS analysis shows that annealing of vanadium oxide films for 30 min in air resulted in a reduction of V_2_O_4.93_ to V_6_O_14_ as a result of the generation of more oxygen vacancies. This reduction causes a decrease of V_oc_ and, thus, the PCE of OPV device. Nevertheless, OPV results confirm that s-V_2_O*_x_* thin film is suitable for optoelectronic devices which are fabricated at high temperatures, and is promising for efficient OPV with a low cost manufacturing process.

## Figures and Tables

**Table 1:** Summary of solar cell parameters with s-V_2_O*_x_* buffer layer annealed at different temperatures for different periods of time.

Ann. Temp.	Maximum PCE (%)	Average PCE_(av)_ (%)	Voc (V)	J_sc_ (mA·cm^−2^)	FF (%)	R_s_ (Ω·cm^2^)	R_sh_ (Ω·cm^2^)
Non	6.0	5.8 ± 0.14	0.86	10.0 ± 0.22	67.1 ± 1.2	11.6 ± 0.9	1264 ± 91
100	6.0	5.9 ± 0.14	0.86	10.1 ± 0.13	68.3 ± 1.7	10.5 ± 1.0	1282 ± 215
200	6.3	5.9 ± 0.26	0.87	10.2 ± 0.14	67.1 ± 2.6	10.3 ± 0.7	1346 ± 246
300	5.6	5.3 ± 0.28	0.83	9.9 ± 0.41	64.3 ± 2.7	12.6 ± 1.7	1095 ± 238
400	5.2	5.0 ± 0.17	0.83	10.1 ± 0.12	59.6 ± 0.9	17.6 ± 0.4	933 ± 84

**Table 2:** Summary of solar cell parameters with s-V_2_O*_x_* buffer layer annealed at different temperatures for different periods of time.

Annealing Temperature	V^4+^ Oxidation State	V^5+^ Oxidation State	Work Function (eV)	Valence Band (eV)	*E_g_* (eV)
Unannealing	15%	85%	5.26	2.53	2.5
200 °C	22.5%	77.5%	5.22	2.53	3.78
300 °C	27%	73%	5.23	2.50	2.68
400 °C	33%	67%	5.19	2.50	2.42
